# Assessment of genome evolution in *Bifidobacterium adolescentis* indicates genetic adaptation to the human gut

**DOI:** 10.1128/msystems.01173-25

**Published:** 2026-02-06

**Authors:** Emanuele Selleri, Chiara Tarracchini, Silvia Petraro, Leonardo Mancabelli, Christian Milani, Francesca Turroni, Yan Shao, Hilary P. Browne, Trevor D. Lawley, Douwe van Sinderen, Marco Ventura, Gabriele Andrea Lugli

**Affiliations:** 1 Laboratory of Probiogenomics, Department of Chemistry, Life Sciences, and Environmental Sustainability, University of Parma 546768, Parma, Italy; 2 Department of Medicine and Surgery, University of Parma 478519, Parma, Italy; 3 Microbiome Research Hub, University of Parma 9370, Parma, Italy; 4 Host-Microbiota Interactions Laboratory, Wellcome Sanger Institute 47665, Hinxton, United Kingdom; 5 APC Microbiome Institute and School of Microbiology, Bioscience Institute, National University of Ireland, Cork, Ireland; Cleveland Clinic, Cleveland, Ohio, USA

**Keywords:** bifidobacteria, genomics, metagenomics, microbiome

## Abstract

*Bifidobacterium adolescentis* is one of the most frequently encountered bifidobacterial species present in the adult human gut microbiota, with a prevalence of approximately 60%. Despite its high prevalence, *B. adolescentis* has not been extensively studied and characterized, and our understanding of its physiological traits, genetic diversity, and potential interactions with other members of the human gut microbiota or with its host is therefore fragmentary. In the current study, a data set comprising 1,682 *B. adolescentis* genomes was compiled by combining publicly available data and metagenome assemblies from 131 projects to uncover the unique genetic characteristics of this species. A pangenome analysis of *B. adolescentis* identified 203 clusters of orthologous genes absent from the other five human-associated *Bifidobacterium* species, six of which were *in silico* predicted to encode functions unique to this taxon. Furthermore, 2,597 genes were predicted to have been acquired by horizontal gene transfer, including genes encoding extracellular structures involved in interaction with the host and other microorganisms, and phage defense mechanisms against bacteriophages. Detailed phylogenetic analysis revealed seven clusters within the *B. adolescentis* species, each partially associated with the origin of strain isolation, suggesting phylogenetic differentiation shaped by geographical strain origin. Moreover, a large-scale metagenomic analysis of over 10,000 human gut metagenomes from healthy adults revealed that *B. adolescentis* co-occurs with 36 putative beneficial commensals and butyrate-producing taxa, highlighting its role as a key bifidobacterial species involved in microbial networking within the adult human gut microbiota.

**IMPORTANCE:**

To comprehensively explore the biodiversity within a microbial species, the reconstruction of a substantial number of genomes is essential. In this study, we successfully uncovered the genetic diversity of *Bifidobacterium adolescentis* by retrieving a large number of genomes from human gut metagenomic samples. The complete overview of the *B. adolescentis* pangenome enabled us to investigate the genetic features that distinguish this gut commensal from other bifidobacterial species residing in the human intestinal microbiota.

## INTRODUCTION

*Bifidobacterium adolescentis* belongs to the genus *Bifidobacterium*, which is a group of gram-positive, high G+C, non-motile, non-spore-forming, and generally obligate anaerobic bacteria belonging to the phylum Actinomycetota. Members of this genus are key components of the gut microbiota in humans and other animals, where they contribute to host health by modulating immune functions, interacting with other intestinal microbes, and helping to prevent dysbiosis (1, 2). Among bifidobacteria, *B. adolescentis* is one of the most relevant species associated with the adult human gut, together with *Bifidobacterium bifidum*, *Bifidobacterium breve*, *Bifidobacterium catenulatum*, *Bifidobacterium dentium*, and *Bifidobacterium longum*, playing an important role in the stability and functional maturation of the intestinal ecosystem (3, 4).

*B. adolescentis* has been detected in the gut microbiome of over 60% of healthy adults (5), representing a prevalence that may be linked to its ability to metabolize a wide range of plant-derived glycans, including starch (5, 6). Scientific evidence indicates that *B. adolescentis* strains can influence other members of the gut microbiota by stimulating the growth of other bifidobacteria and increasing the relative abundance of butyric acid-producing microorganisms, such as *Eubacterium* spp., *Roseburia* spp., and *Butyrivibrio* spp. (7–9). Furthermore, *B. adolescentis* has been proposed to exhibit protective activity in the gut environment by preventing colonization of potentially pathogenic species or controlling the levels of bacteria that may disrupt the eubiotic state (10, 11). Additional beneficial interactions with the host have been reported in recent years, such as the ability to improve the intestinal barrier by increasing mucin production, strengthening tight junctions (TJ), reducing systemic permeability and inflammation, and inducing immune regulatory responses (5).

*B. adolescentis* is known to interact with the host immune system through the production of short-chain fatty acids (SCFAs), which support intestinal health by positively influencing TJ proteins and promoting intestinal wall repair. Certain *B. adolescentis* strains have been reported to elicit beneficial effects in the prevention and alleviation of symptoms associated with intestinal diseases and disorders, including inflammatory bowel disease, irritable bowel syndrome, and colorectal cancer. These effects are primarily mediated by this bacterium’s ability to strengthen the intestinal barrier and modulate key enzymes involved in tumor progression (12–14). However, despite these promising observations, the molecular mechanisms underlying such effects remain poorly defined, and the genetic determinants responsible for strain-specific properties are largely unknown. Additionally, the ecological role of *B. adolescentis* within the gut microbial community, including its interactions with other commensals and the host, remains incompletely understood.

Due to the absence of a comprehensive pangenome analysis, it remains unclear which genetic determinants are characteristic of *B. adolescentis* and what factors underlie its high prevalence within the human gut microbiota. To address this gap, the present study investigates the biodiversity of the species by analyzing its genetic and metabolic repertoire. For this purpose, a data set of 1,682 genomes of *B. adolescentis* was established, which was then used to assess the genetic and functional potential of this species. In addition, metagenome-driven investigation allowed examination of the global distribution and ecological interactions of *B. adolescentis* across 10,620 gut microbiomes of healthy adults, highlighting its geographically structured prevalence and its association with beneficial commensal and butyrate-producing bacteria.

## RESULTS

### *B. adolescentis* pangenome and general genome features

**Fig 1:**
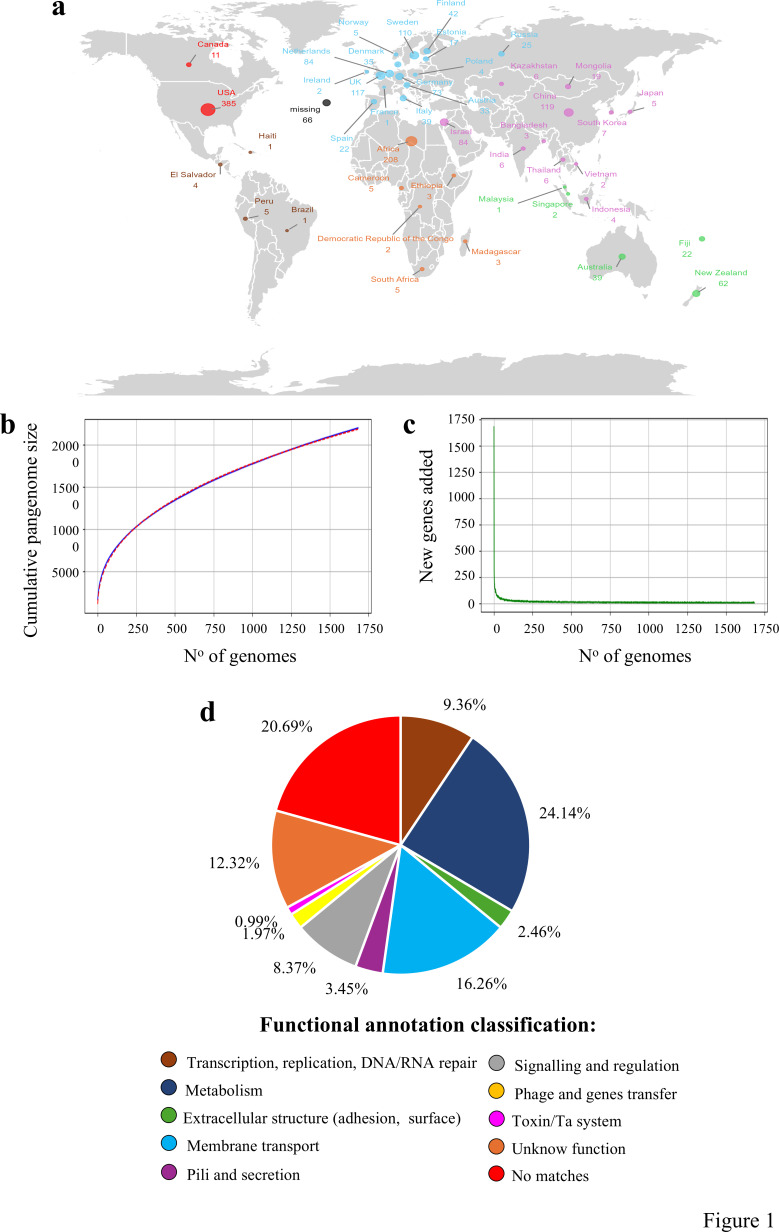
Worldwide distribution and pangenome architecture of *B. adolescentis*. Panel **a** exhibits the distribution of the *B. adolescentis* genomes based on their geographical origin. Each country is represented by a circle whose color indicates the macro-region and whose size reflects the number of analyzed genomes. Panel **b** illustrates the trend of the pangenome, represented by variations in the size of the resulting gene pool upon the sequential addition of the 1,682 *B. adolescentis* genomes. The *x*-axis represents the number of included genomes, whereas the *y*-axis represents the number of genes in the generated pangenome. In a similar fashion, panel **c** shows the average of new genes upon sequential addition of the *B. adolescentis* genomes. Panel **d** displays the percentages of functional annotations identified for the unique clusters of orthologous genes (COGs) of *B. adolescentis* compared to other bifidobacterial species colonizing humans. Predicted functions of different COGs are highlighted in different colors.

To explore the biodiversity of the species *B. adolescentis* as comprehensively as possible, genomic sequences were collected from 131 genomic and metagenomic projects. Among genomes of isolated strains and metagenome-assembled genomes (MAGs), 2,092 genomes were classified as high-quality genomes by CheckM2 (15). These genomes were subsequently dereplicated using dRep (16), resulting in a final high-quality data set composed of 1,201 unique MAGs and 481 genomes of isolated *B. adolescentis* strains. These 1,682 genomes, representing *B. adolescentis* strains originating from 44 countries (Fig. 1a), were predominantly of human origin (99.95%), with only a small fraction (0.05%) derived from other animal hosts. Among them, 879 genomes were reconstructed within the framework of this project, 42 from biological isolates and 837 as MAGs, thereby substantially expanding the currently known genetic diversity of this bifidobacterial species (Table S1). A preliminary screening revealed an average genome size of 2.16 Mb, a GC content of 59.5%, and a predicted set of 1,778 coding sequences (CDS), 54 tRNA genes, and four rRNA loci (Table 1).

**TABLE 1:** General genome features of human-associated *Bifidobacterium* species

Feature	*B. adolescentis*	*B. bifidum*	*B. breve*	*B. catenulatum*	*B. dentium*	*B. longum*
Number of genomes	1,682	512	344	169	129	2,173
Bases (Mb)	2.16 ± 0.15	2.19 ± 0.2	2.39 ± 0.29	2.12 ± 0.23	2.49 ± 0.28	2.37 ± 0.21
CDS	1,778 ± 148	1,826 ± 170	2,033 ± 312	1,792 ± 216	2,072 ± 210	1,991 ± 195
tRNA	54.4 ± 5	52.1 ± 6.3	53.7 ± 4.3	51.8 ± 8.2	52.9 ± 8.5	57.6 ± 9.5
rRNA loci	4	2.8	2.6	5	4	3.8
GC content	59.5 ± 0.3	62.7 ± 1	58.9 ± 2.4	55.9 ± 1.1	58 ± 3.3	59.9 ± 0.7
Soft-core COGs	1,162	1,243	1,410	646	339	1,081
Carbohydrate-active enzymes (CAZymes)	120.95 ± 11.67	99.43 ± 8.1	124.35 ± 6.69	135.82 ± 7.1	166.36 ± 7.6	126.2 ± 8.2
CAZymes families	61	61	48	52	80	43
Transposase	10.5 ± 4.7	23.7 ± 8.3	32.2 ± 14.3	13.5 ± 9.2	7 ± 5.4	28.8 ± 14.9
Bacteriocin prevalence	24%	78%	16%	21%	78%	34%
Prophage prevalence	46%	47%	26%	34%	58%	29%
CRISPR locus prevalence	50%	37%	67%	72%	78%	40%

The inclusion of genomes from metagenomic projects resulted in the reconstruction of a closed pangenome (Fig. 1b), indicating that the genetic diversity of *B. adolescentis* has been fully captured. In contrast, reconstruction of the pangenomes of the other five species commonly found with the human host, that is, *B. bifidum*, *B. breve*, *B. catenulatum*, *B. dentium*, and *B. longum*, showed that their pangenomes are still open, suggesting that their overall genetic diversity has yet to be fully characterized due to the limited number of publicly available genomes (Fig. S1) (17).

Dissection of the *B. adolescentis* pangenome revealed the presence of 1,162 soft-core clusters of orthologous genes (COGs), that is, representing genetic functions identified in at least 95% of the genomes in the data set. Among the 1,778 *B. adolescentis* CDS, 10 ± 5 were associated with transposases and 121 ± 12 with the glycobiome (spanning 61 families). Additionally, their distribution enabled the identification of prophage sequences in 46% of the genomes and CRISPR loci in 50% of them (Table 1). When comparing the general genome features of *B. adolescentis* with those of the other five human-related bifidobacterial taxa, we observed that this species exhibited the lowest number of CDS (Table 1). Accordingly, none of the analyzed genomic features appears to be more represented in *B. adolescentis* than in the other bifidobacteria. On the contrary, it displayed the second-smallest genome size after *B. catenulatum* (2.12 ± 0.23 Mb), which is the second-lowest number of glycobiome-related enzymes after *B. bifidum* (99 ± 8), and the second-lowest number of transposases after *B. dentium* (7 ± 5) (Table 1).

### Prediction of *B. adolescentis*-specific functional traits

To identify functions unique to the *B. adolescentis* taxon, a comparative genomic analysis was performed, including all currently identified bifidobacterial species (Table S2), with the specific aim of investigating taxa associated with the human host. Comparison of *B. adolescentis* COG sequences with those of the other five human-associated bifidobacterial species led to the identification of 203 unique COGs exclusively present in the *B. adolescentis* soft-core genome (Table S3).

*In silico* functional categorization of the 203 COGs showed that approximately 25% of the genes have annotations or domains corresponding to transporters, proteins required for sortase-dependent pilus biosynthesis, and functions associated with adhesion and the secretion of extracellular proteins (Fig. 1c). These functional predictions, derived from homologous protein alignments and conserved domain searches, suggest that *B. adolescentis* possesses a conserved species-specific gene set involved in the colonization and interaction with the human gut. Analysis of COG synteny further revealed that 38 predicted genes form seven genetic clusters distributed across the *B. adolescentis* genomes. Two of these gene clusters contain loci involved in modifying the cell envelope to enhance host interaction, including a putative Tad pilus locus and enzymes associated with cell wall remodeling and teichoic acid biosynthesis (Table S4). These features have been directly linked to colonization capacity and long-term persistence in the gut, as demonstrated in previous studies (18, 19). The genes found within the remaining five loci reflect additional hallmarks of intestinal adaptation, including carbohydrate metabolism via a β-galactosidase, tolerance to acidic microenvironments, metal ion detoxification, a competence island enabling efficient DNA uptake, and transport systems linked to folate-related metabolites (Table S4). Together, these metabolic and stress-response traits point to a physiological profile that is finely attuned to the ecological conditions of the human gut (20–23).

Digging deeply into the unique COG domain sequences, six of them showed high sequence homology to the genes *all*B, *cnt*D, *deg*U, *feo*B, *tsa*D, and *php*P, each displaying predicted functions unique to *B. adolescentis* and absent in the other human-associated species of the genus. Four of the latter genes were associated with interaction mechanisms with the extracellular environment. More specifically, *feo*B and *cnt*D encode metal ion transporters, *tsa*D is involved in stress resistance, and *deg*U regulates genes responsible for the production of biofilm and degradative enzymes (24–26). The other two COGs, *php*P and *all*B, have distinct roles, such as regulating DNA transcription via dephosphorylation and purine degradation, respectively.

Several studies have identified *B. adolescentis* as a microorganism involved in the stabilization of the intestinal microbiota, thereby contributing to the prevention of intestinal diseases (12, 13, 27, 28). The presence of COGs identified as unique to the species, with functional domains related to adhesion, membrane transport, and regulation, may be one of the factors that allows the species to colonize the intestine and respond quickly to environmental changes in its ecological niche. Additionally, the identification of functional domains related to the production of cofactors and carbohydrates suggests how the species produces bioactive molecules that modulate host immunity, strengthen the intestinal barrier, and reduce inflammatory events.

### Ecological dissection of the *B. adolescentis* species

**Fig 2:**
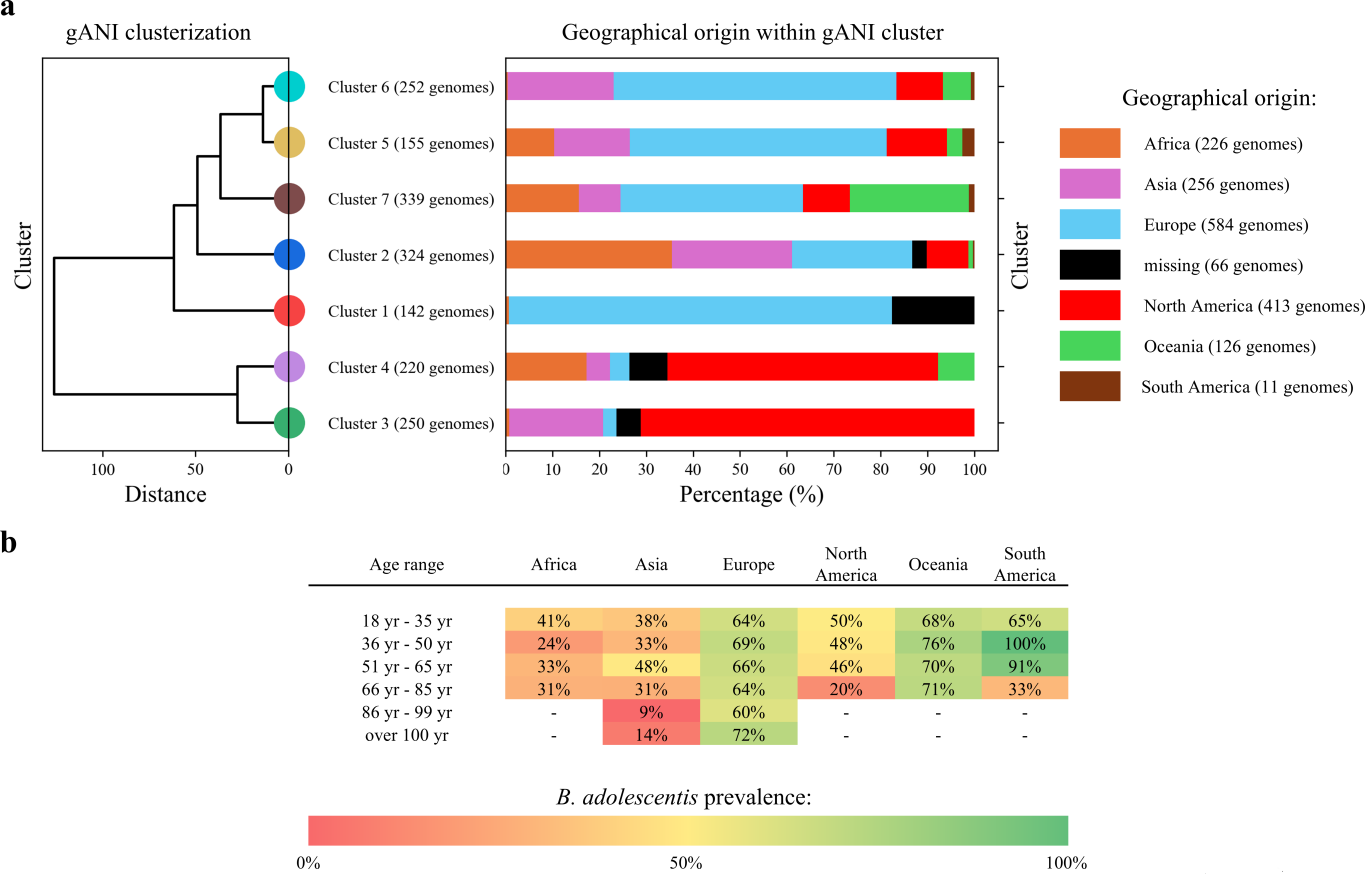
Correlation between *B. adolescentis* genotypes and their geographic origin. Panel **a** on the left shows a dendrogram derived from the HCA of 1,682 *B. adolescentis* genomes, based on the gANI matrix used to explore the genome biodiversity. The number of genomes within each cluster is reported below the corresponding cluster label. On the right, the geographical distribution within each cluster is displayed as relative abundances. Different colors in the histogram indicate the geographical origin of the genomes. Panel **b** shows the prevalence of *B. adolescentis* in the 10,620 metagenomic samples, stratified by both geographic region and age range.

To explore the genome diversity within *B. adolescentis*, a phylogenomic analysis was performed using the chromosomal sequences of the 1,682 dereplicated genomes. A hierarchical clustering analysis (HCA) was carried out based on the genomic average nucleotide identity (gANI) across the entire data set. This method identified seven distinct phylogenomic clusters, revealing substantial genomic divergence among the analyzed genomes, as determined by applying the Elbow Method to the intra-cluster sum of squares (Fig. 2a). The genetic distances among clusters were further evaluated, revealing comparable ANI ranges and median values across each cluster, except for Cluster 3, which showed lower variability and the highest median ANI value of 98 (Fig. S2). Overall, these data do not support the existence of distinct sub-lineages within the species, even after the inclusion of *B. adolescentis* synonyms, such as *Bifidobacterium stercoris* and *Bifidobacterium faecale*, which were previously misclassified as separate taxa (29, 30).

The geographical origin of the genomes from any of the considered macro-regions (Africa, Asia, Europe, North America, South America, and Oceania) was evaluated, revealing several statistically significant correlations between clusters and geographical origin (significance thresholds of *P*-value < 1 × 10^−5^ and FDR < 0.05). In detail, Cluster 2 was associated with the African region (35.49% of its genomes) and Asia (25.62%), while further statistically significant correlations were observed between Clusters 1 and 7 with Europe and Oceania, respectively, and between Clusters 3 and 4 with North America (Fig. 2a).

Besides geographical patterns, we also examined whether the distribution of *B. adolescentis* was associated with the age of the host. From a metagenomic data set comprised of 10,620 fecal samples of healthy adults collected across 65 different BioProjects (Table S5), 3,020 samples for which host age was available were included in this analysis. Individuals aged 18 to over 100 years were grouped into six age classes, and the prevalence of *B. adolescentis* was assessed across these groups. Once again, the presence of *B. adolescentis* appeared to be driven more by geographical origin than by age (Fig. 2b). Regardless of host age, Europe and Oceania consistently showed the highest prevalence of *B. adolescentis* (from 60% in Europe in the 86- to 99-year-old group to 76% in Oceania in the 36- to 50-year-old group), whereas Africa and Asia displayed the lowest prevalence (from 9% in Asia in the 86- to 99-year-old group to 48% in Asia in the 51- to 65-year-old group).

The phylogenomic and metagenomic analyses thus revealed distinct *B. adolescentis* clusters associated with their geographic origin, indicating that in adults, the distribution of this species is primarily driven by geography rather than host age.

### Global distribution of *B. adolescentis* and its correlation with other members of the human gut microbiota

**Fig 3:**
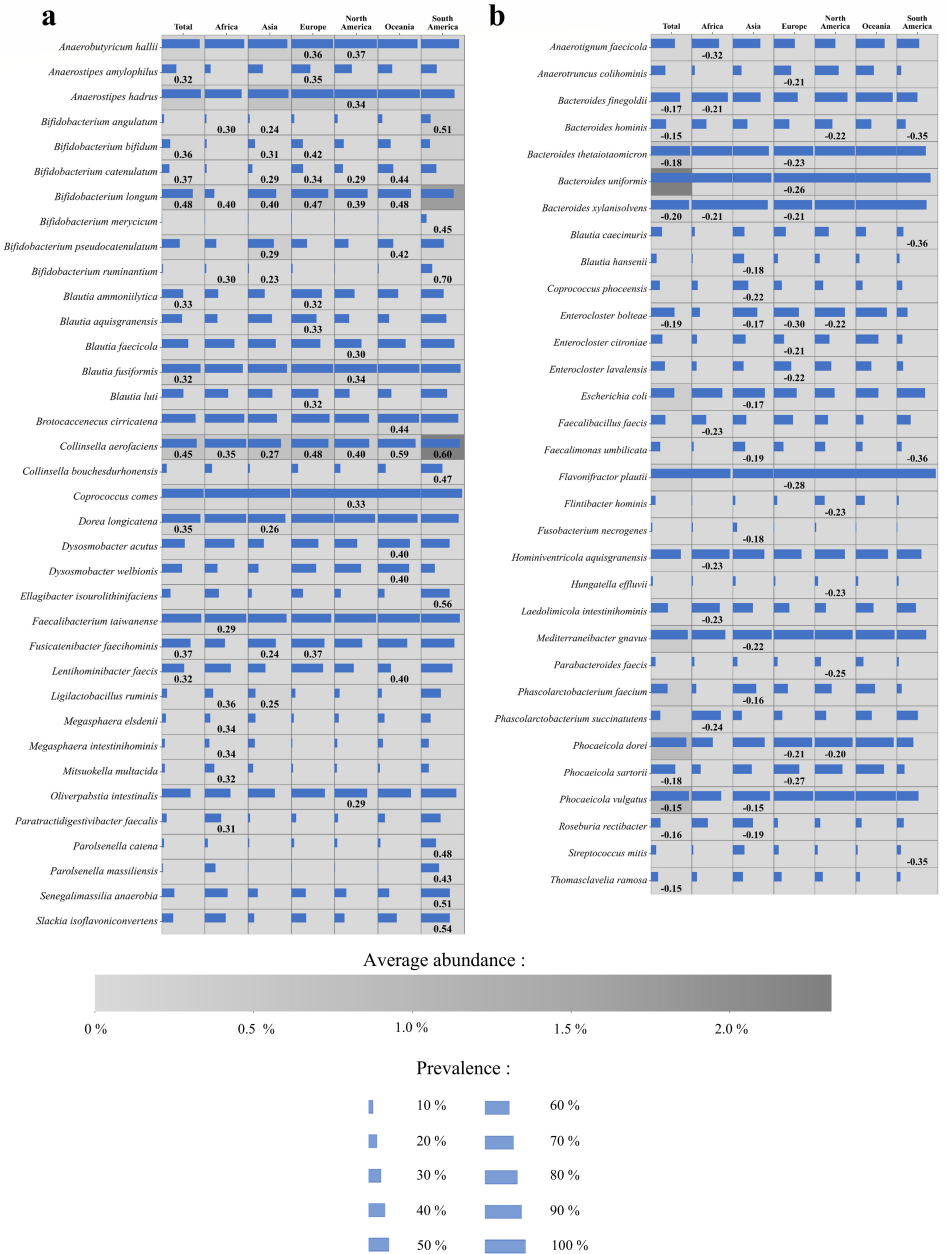
Correlations of *B. adolescentis* with other microbial species of the human gut microbiota. Panel **a** displays the species exhibiting the highest positive correlations with *B. adolescentis* in at least one macro-region. The first column represents correlations across the entire data set, which comprises 10,620 metagenomic samples, while the remaining columns correspond to specific macro-regions, each highlighted by its respective color. Within cells, the horizontal purple bar indicates the prevalence of the species in the data set, while the background color reflects its mean abundance. Similarly, panel **b** shows the species that have the highest negative correlations with *B. adolescentis*.

Given the geographic structure observed in the ANI-based phylogenetic clusters, we next assessed how geographical origin influences the associations between *B. adolescentis* and other bacterial taxa within the adult gut microbiota by analyzing and comparing metataxonomic profiles (31). To assess the co-occurrence of *B. adolescentis* with other gut microbes, we performed a correlation analysis across different geographic regions using the microbiota profiles of 10,620 fecal samples of healthy adults (Table S5), identifying positive correlations with 226 microbial species and negative correlations with 114 species (significance thresholds of *P*-value < 1 × 10^−5^ and FDR < 0.05) (Table 2; Table S6). Among positively correlated species, *B. longum* and *Collinsella aerofaciens* were shown to exhibit significant associations across all geographic regions, indicating stable and conserved ecological relationships, which may involve functional cohabitation, interdependent metabolic activities, or mechanisms of coevolution (Fig. 3a). Additionally, several of the positive correlations involved bacterial species already recognized as beneficial in literature, including *Akkermansia muciniphila*, *Intestinimonas butyriciproducens*, *Mediterraneibacter butyricigenes, Faecalibacterium prausnitzii*, *Agathobacter rectalis*, and *Lactobacillus acidophilus* (32–37). These taxa are known for their anti-inflammatory properties and the production of metabolites promoting epithelial integrity, stimulating the immune system, and overall gut health. More specifically, while species like *Akkermansia muciniphila* were found to be associated with the entire data set (significance thresholds of *P*-value < 1 × 10^−5^ and FDR < 0.05), several associations with butyrogenic bacteria were geographically specific (Fig. 3a; Table S6). For example, *Blautia* spp. have been found to be positively associated in Europe and North America, while *Coprococcus* and *Ruminococcus* spp. have been found in Asia, Europe, and North America. In contrast, *Faecalibacterium* spp. was found to co-occur with *B. adolescentis* in all macro-regions except Oceania (38–40). It is well established that *B. adolescentis* ferments oligosaccharides, releasing acetate, a metabolite in turn utilized by butyrate-producing bacteria (5). The correlation between *B. adolescentis* and butyrogenic bacteria identified in different geographical origins suggests cross-feeding mechanisms with geographically specific microbial partners.

**TABLE 2:** Correlation of six *Bifidobacterium* species within 10,620 gut metagenomes of healthy adults

Species	Average abundance	SD abundance	Prevalence	Correlation	Positive correlation	Negative correlation
*Bifidobacterium adolescentis*	1.04%	2.81%	54.03%	340	226	114
*B. adolescentis* Africa region	0.22%	0.77%	33.33%	31	24	7
*B. adolescentis* Asia region	0.77%	2.55%	36.89%	111	79	32
*B. adolescentis* Europe region	1.39%	2.90%	69.82%	204	122	82
*B. adolescentis* North America region	0.52%	1.36%	55.74%	47	41	6
*B. adolescentis* Oceania region	0.95%	2.48%	63.64%	8	8	0
*B. adolescentis* South America region	2.97%	6.11%	76.65%	32	28	4
*Bifidobacterium bifidum*	0.19%	0.85%	19.60%	202	138	64
*Bifidobacterium breve*	0.03%	0.45%	7.11%	280	116	164
*Bifidobacterium catenulatum*	0.07%	0.43%	17.90%	263	172	91
*Bifidobacterium dentium*	0.02%	0.26%	8.31%	302	235	67
*Bifidobacterium longum*	0.86%	2.86%	71.21%	416	272	144

In contrast, negative correlations were predominantly observed among species belonging to the Bacteroidota phylum, including 26 species of *Bacteroides*, seven *Phocaeicola*, and six *Parabacteroides* (Table S6). From a geographic perspective, these taxa were negatively associated with *B. adolescentis* in Europe and North America, with *Phocaeicola* and *Bacteroides* also showing negative associations in Asia, and *Bacteroides* in South America. Furthermore, negative correlations were observed with pathogenic or opportunistic bacteria, such as species of the genera *Citrobacter*, *Enterobacter*, *Escherichia, Fusobacterium,* and *Klebsiella,* whose presence is associated with the onset of various gut diseases, including tumorigenesis (41–44). In a few cases, the negative correlations displayed geographical specificity with pathogenic or opportunistic bacteria, likely reflecting the fact that the metagenomic data set included only healthy adults, thereby reducing the prevalence of opportunistic pathogens across microbiomes. Nevertheless, specific negative correlations were observed with *Escherichia* spp. in Asia, *Clostridium symbiosum* in Asia and Europe, and *Streptococcus mitis* in South America, suggesting a degree of geographic specificity among taxa with opportunistic or pathogenic potential (45, 46).

**Fig 4:**
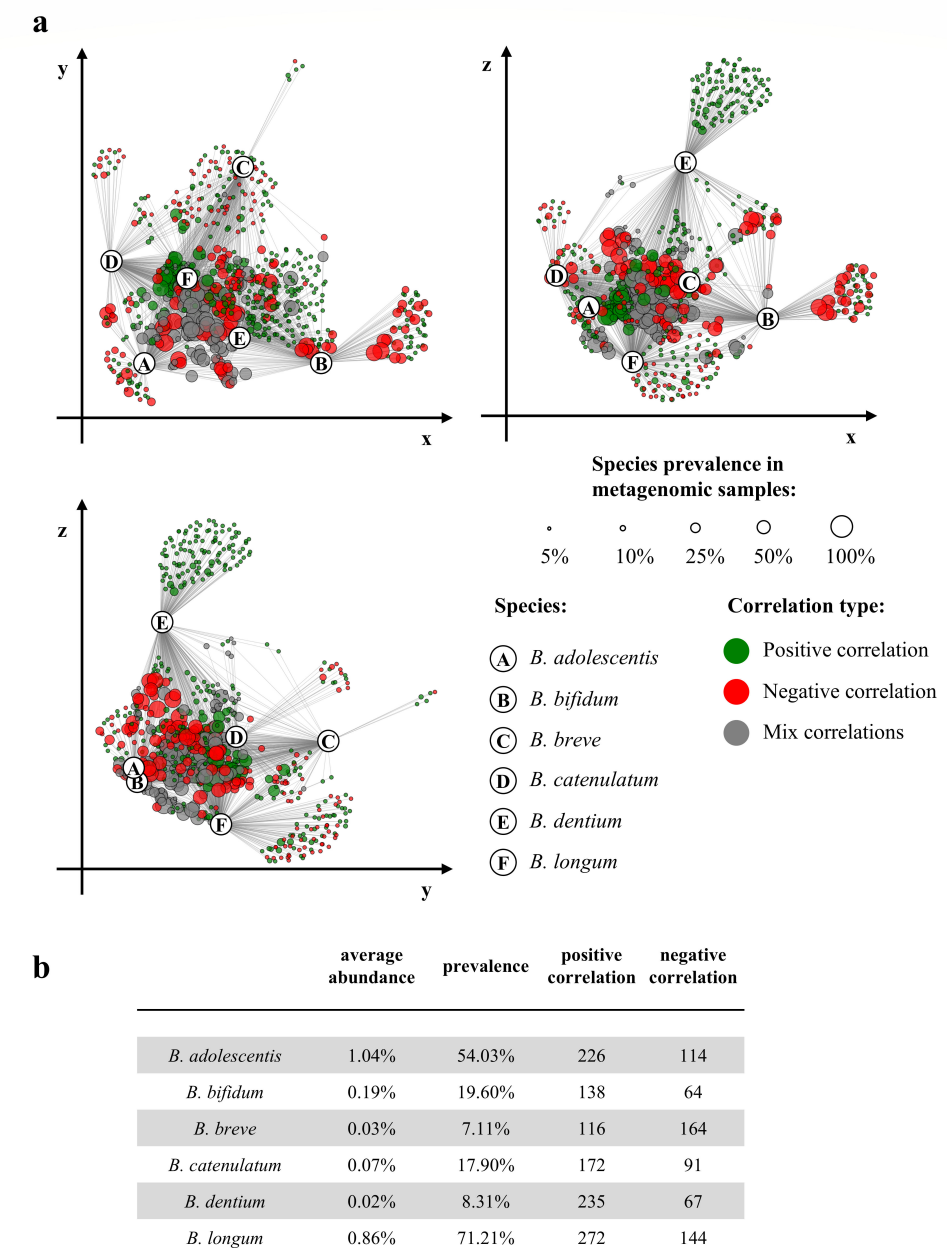
Network of human-associated *Bifidobacterium* species within the gut microbiome. Panel **a** depicts microbial interaction networks among members of the adult gut microbiota. Each node represents a bacterial species, with node size proportional to its prevalence across metagenomic samples and colors indicating the direction of association (green: positive; red: negative). The six human-associated *Bifidobacterium* species are highlighted in distinct colors. Panel **b** summarizes the abundance, prevalence, and number of correlations for these species.

To compare the co-existence of *B. adolescentis* with that of other human-associated species of the *Bifidobacterium* genus, their co-occurrence with other members of the adult gut microbiota was evaluated. *B. adolescentis* was shown to elicit 340 significant co-occurrence correlations, followed only by *B. longum*, which was shown to exhibit 416 interactions with other gut microbes, indicating that *B. adolescentis* is among the most interactive *Bifidobacterium* species within the human intestinal microbiota (Table 2; Fig. 4).

From our results, *B. adolescentis* appears to be associated with a wide range of microbial partners, suggesting that it engages in interactions, co-metabolism, and/or cross-feeding strategies that differ from those of other *Bifidobacterium* species occupying the same ecological niche.

### Pangenome plasticity and accessory genes of *B. adolescentis* species

**Fig 5:**
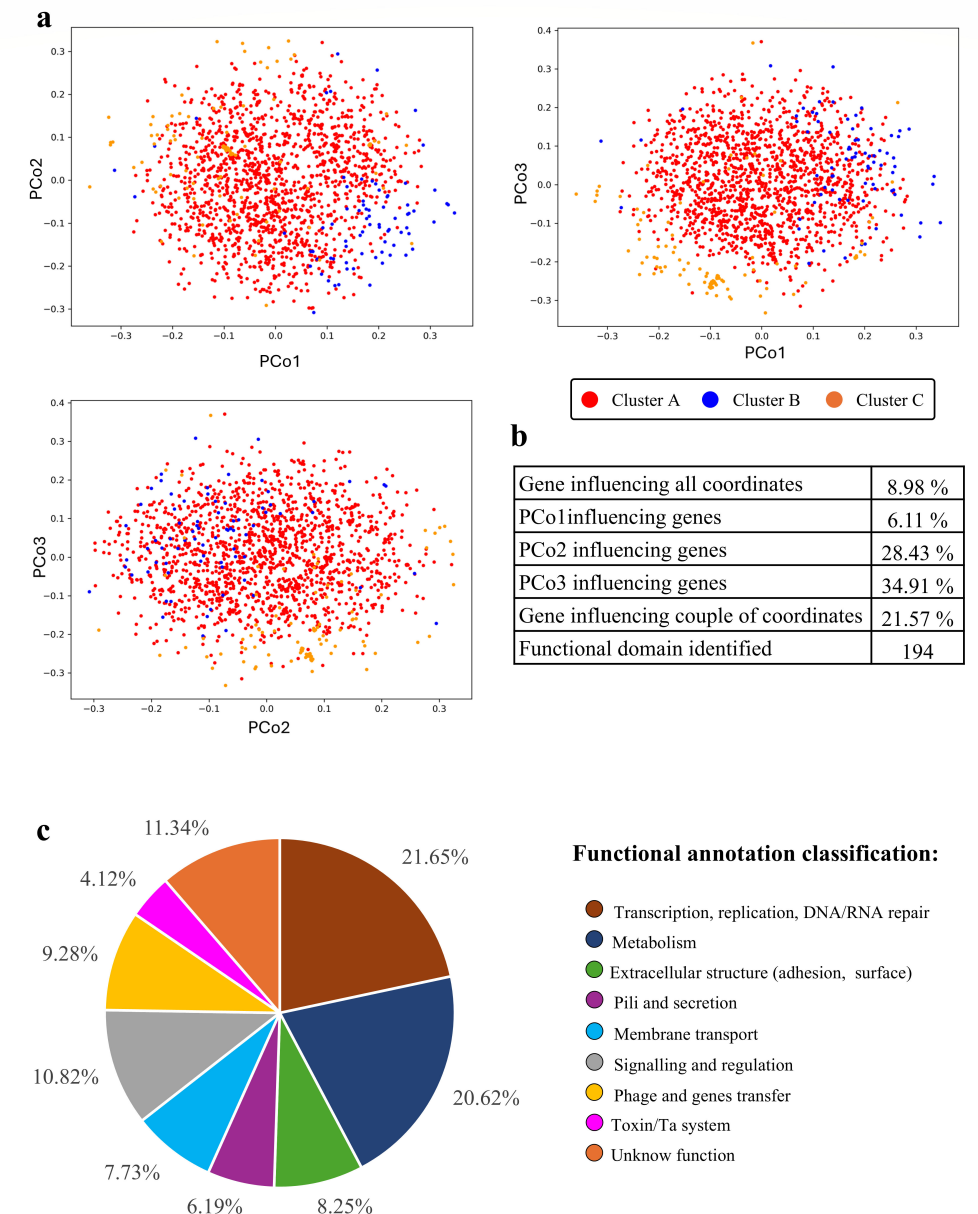
Accessory genome of the *B. adolescentis* species. Panel **a** shows the principal coordinates analysis (PCoA) plotted across pairs of coordinates, highlighting three significant clusters represented in different colors. Panel **b** reports the percentages of COGs influencing the PCoA coordinates. Panel **c** illustrates the distribution of predicted functional annotations identified in the accessory genome of *B. adolescentis*. Predicted *in silico* functions are highlighted in different colors.

To explore the genetic diversity of *B. adolescentis*, the 12,774 accessory genes were clustered based on their presence-absence patterns across the 1,682 genomes, revealing three statistically supported groups as identified by the Elbow method and HCA analysis (Fig. 5a).

A subset of accessory genes (6.28% of the analyzed COGs) was found to significantly influence genome distribution in the principal coordinates analysis (PCoA) space. Some of these genes were shown to affect all three principal coordinate dimensions, while others were associated with specific axes or combinations of axes (Fig. 5b). Protein domain analysis of this subset revealed a wide range of functional categories, underscoring their contribution to genome-level variability and the resulting clustering patterns. Among them, 20% of the genes influencing the PCoA displayed functional annotations linked to membrane systems and adaptation to extracellular conditions. Domains related to cell adhesion and secretion accounted for a considerable number of genes, that is, 50 and 57 genes, respectively (Fig. 5c). These findings suggest that variability in the accessory genome is confined to surface structures and environmental responsiveness.

The presence of these functionally annotated genes suggests that the accessory genome of *B. adolescentis* may be modified by selective environmental pressures. Combined with the existence of a stable, species-specific set of core and soft-core genes, these results indicate that while the organism has likely acquired essential traits for niche colonization, environmental factors may continue to shape its accessory genome.

### Horizontal gene transfer (HGT) shaped the genomes of the *B. adolescentis* taxon

**Fig 6:**
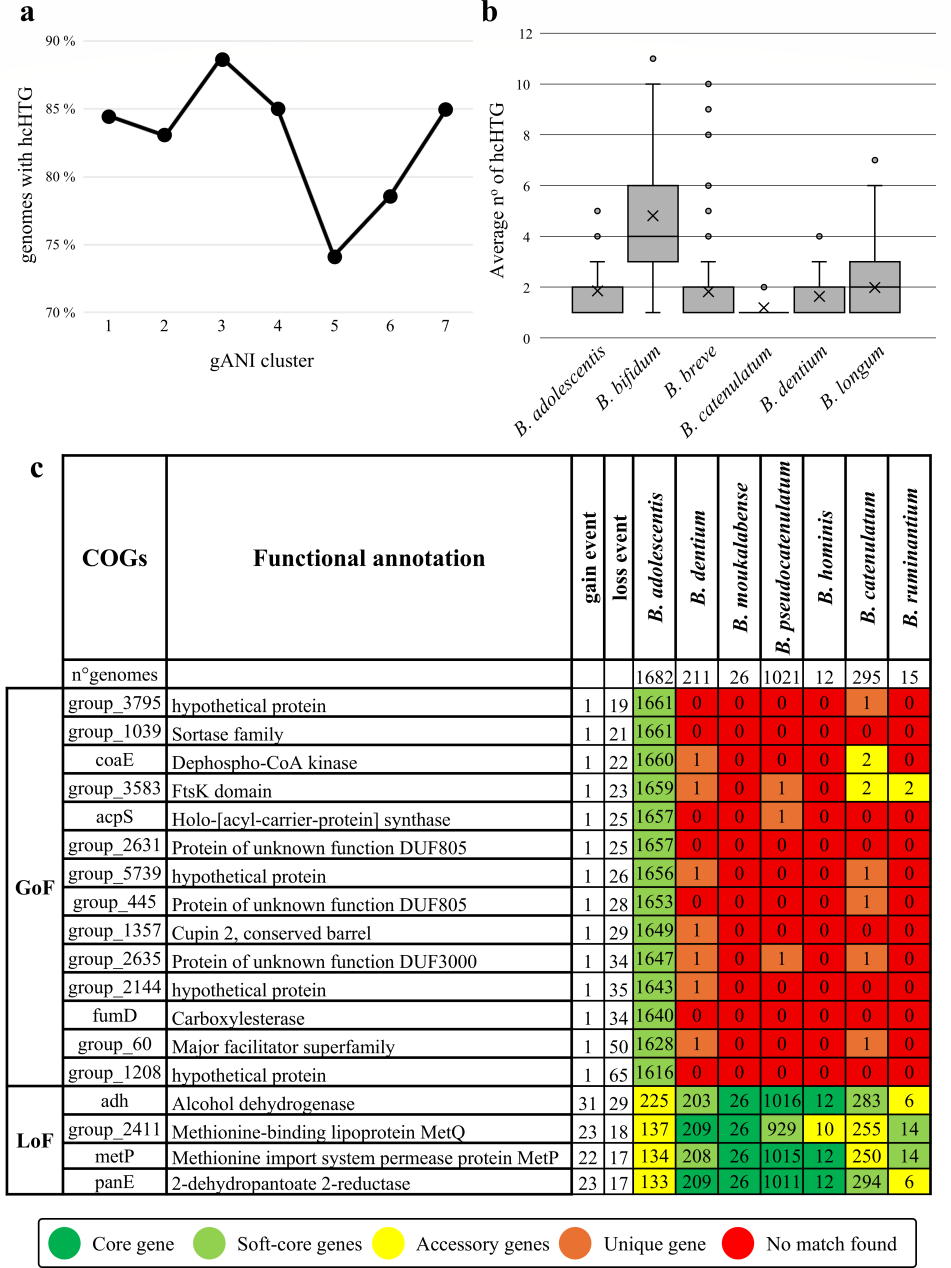
Genome modification events of the *B. adolescentis* taxon. Panel **a** illustrates the number of predicted hcHTGs identified across the genomes belonging to the seven gANI clusters. Panel **b** depicts a box-and-whisker plot representing the distribution of hcHTGs among *Bifidobacterium* species that colonize humans. The *y*-axis shows the number of putative hcHTGs, while species are reported on the *x*-axis. Boxes represent 50% of the data set (distributed between the first and third quartiles), the central X denotes the mean, whiskers indicate the range, and dots represent outliers. Panel **c** exhibits the number of COGs exhibiting significant GoF or LoF events in the pangenome of the species belonging to the *B. adolescentis* group, with different color patterns indicating gene presence across pangenomes.

To comprehensively assess genome variability, COGs potentially acquired through HGT were identified by screening for codon usage bias (CUB) and GC content, revealing 233,949 genes in the pangenome as putative HGT-derived elements. Among them, 2,597 genes were classified as high-confidence horizontally transferred genes (hcHTGs), as they tested positive across all parameters used in the screening (see Materials and Methods), indicating their reliable identification as HGT-derived elements. Examining mobile elements within a 2,000-nt window, we found that 26% of these genes were associated with transposases, supporting their putative mobilizable nature and the involvement of genetic elements that may mediate their transposition. In the *B. adolescentis* data set, hcHTGs were detected in 1,399 genomes (83.71%) (Table S7). Although these genes showed no evident correlation with geographical origin, they displayed significant association with specific genomic clusters (significance thresholds of *P*-value < 1 × 10^−5^ and FDR < 0.05), particularly Clusters 3 and 5, which contained a higher number of genomes carrying hcHTGs (Fig. 6a).

Interestingly, among the putative donor taxa predicted to be responsible for the identified HGT events, four species emerged as major contributors, namely, uncultured *Mobiluncus* sp., *Massiliimalia timonensis*, *Collinsella aerofaciens*, and *Collinsella ihumii* (Table S7). All predicted donors were gram-positive anaerobic bacteria inhabiting the human gut microbiota, suggesting that these HGT events likely occur in the gut environment. With the exception of *Collinsella aerofaciens*, the remaining species have been classified only recently (47, 48), and current knowledge of uncultured *Mobiluncus* sp. is derived from MAG-based reconstructions, having been successfully cultivated in the laboratory (49). Taken together, these observations point to the human gut as a plausible ecological hotspot facilitating the observed gene transfer events.

The number of *B. adolescentis* hcHTGs was furthermore compared with that of other human-associated *Bifidobacterium* species (Table S8). *B. adolescentis* showed an average number of hcHTGs similar to that of other assessed bifidobacterial species, except for *B. bifidum,* which showed a significantly higher value (4.82 average genes per genome against 1.75 average genes per genome for the other species) (Fig. 6b). These findings suggest that *B. adolescentis* exhibits a similar gene acquisition frequency and perhaps analogous HGT mechanisms when compared to other human-colonizing species, possibly due to similar selective pressures or conserved niche-adapting gene patterns within the genus.

The *ect*G gene and five hypothetical encoding proteins were hcHTGs identified as part of the species core genome. This finding supports the notion that the latter hcHTGs were the result of ancient species-defining HGT events linked to early speciation rather than more recent gene acquisitions, which typically become part of the accessory genome.

Analysis of the hcHTGs amino acid sequences from the accessory genome revealed that thirteen genes were linked to cellular defense functions, including five with CRISPR-Cas-related domains, such as *cas*9, *cas*2, *cse*3, and *cas*C. Previous studies have highlighted the variability and diversity in the structure of CRISPR-Cas systems among bifidobacterial species, leading to the classification of different system types (50, 51). In these studies, *B. adolescentis* was identified as possessing a Type I-C CRISPR-Cas system, characterized by the presence of cas3, along with the universal genes cas1 and cas2, and accessory genes such as cas4, cas5, cas7, and cas8. Prompted by the presence of these genes, analysis through the CRISPRCasFinder database identified 178 Type I CRISPR-Cas systems, subdivided into 57 I-C, 41 I-E, and 80 I-G, and 98 Type II systems, subdivided into 86 II-A and 12 II-C (Table S9).

The identification of these genes revealed a heterogeneous distribution of CRISPR-Cas systems, also reflected across the seven gANI clusters and the geographical origin of the genomes, with significant associations observed for the I-E system in Clusters 1 and 3, the I-G system in North American isolates, and the II-A system in those from Oceania (*P*-value < 0.05) (Table S9). The screening also retrieved 11,718 unique spacer sequences across 834 *B. adolescentis* genomes. Comparison of these sequences against all NCBI viral genomes revealed a predicted susceptibility toward immunization against phages belonging to the Siphoviridae and Podoviridae families (772 and 401 genomes, respectively). However, 755 *B. adolescentis* genomes harbor spacers associated with viruses that have not yet been classified. Using NCBI-curated phage sequences, unique spacers exhibit higher sequence similarity against *Bifidobacterium* phage BD811P2, *Propionibacterium* phage G4, *Edwardsiella* phage Edno5, *Rheinheimera* phage vB_RspM_Barba21A, and *Ralstonia* phage RSL2 DNA, all phages belonging to the order Caudoviricetes (Table S10).

Together, these results suggest that CRISPR-Cas genes in *B. adolescentis* are likely derived from HGT events and represent non-native elements shaped by both phylogenetic background and environmental factors, with the presence of both Type I and Type II CRISPR-Cas systems detected among the genomes.

### Inter- and intraspecies-specific patterns of gene acquisition and loss in *B. adolescentis*

Building on the previously performed *Bifidobacterium* pangenome analysis, a focused comparison was conducted on the *B. adolescentis* phylogenetic group, comprising *B. catenulatum*, *B. dentium*, *B. hominis*, *B. moukalabense*, *B. pseudocatenulatum*, and *B. ruminatium*, to investigate gain-of-function (GoF) and loss-of-function (LoF) processes specific to *B. adolescentis*.

Within the *B. adolescentis* phylogenetic group, 606 COGs were associated with significant GoF events (significance thresholds of *P*-value < 1 × 10^−5^ and FDR < 0.05), 271 with significant LoF events, and 1,807 were characterized by both evolutionary events (Table S11). The proteome of each genome belonging to the *B. adolescentis* phylogenetic group was then used to validate the GoF and LoF events of the *B. adolescentis* taxon. The screening enabled the identification of 14 COGs in the soft core of *B. adolescentis*, which exhibited significant GoF events compared to the other species. Half of these COGs revealed functional annotations related to metabolism (*coa*E, *acp*S, and *fum*D) and interaction with the extracellular environment (Fig. 6c).

Furthermore, four COGs belonging to the cloud genome of the species (shared in less than 15% of *B. adolescentis* genomes) were identified as highly conserved in other species of the same phylogenetic cluster. The presence of these COGs in the pangenomes of the species belonging to the same phylogenetic group, that is, *B. dentium* and *B. moukalabense*, suggests that they may represent a signature of genetic functions inherited from the common ancestor of the clade. Subsequently, these COGs undergo LoF events within the *B. adolescentis* phylogenetic group, as also observed in the *B. catenulatum* and *B. ruminatium* species. Interestingly, among these LoF COGs, two were predicted to be involved in methionine transport. Overall, the data indicate that *B. adolescentis* has undergone a distinct genetic evolution compared to its closest relatives, acquiring genes associated with metabolic and environmental functions, while losing presumably ancestral functions, such as methionine transport, highlighting a process of ecological specialization.

## DISCUSSION

The results of this comparative genomic analysis provide further confirmation that *B. adolescentis* has evolved to be specialized for colonization and persistence in the human gut microbiota (12, 14, 52). Our analyses revealed several genomic loci that underpin this adaptation. These include operons involved in cell envelope modification, such as a putative Tad pilus locus and enzymes for cell wall remodeling and teichoic acid biosynthesis, contributing to host interaction, adhesion, and long-term persistence. Additional genetic loci encode functions supporting intestinal survival, including β-galactosidase-mediated carbohydrate utilization, tolerance to acidic microenvironments, metal ion detoxification, competence-related genes enabling efficient DNA uptake, and transport systems associated with folate metabolism. Collectively, these loci reflect a conserved species-specific gene repertoire finely tuned to the chemical, nutritional, and ecological conditions of the gut.

Furthermore, a stable and conserved set of COGs with functions related to colonization and persistence in the human intestinal environment was also identified. These include genes involved in adhesion, such as *fts*W and *tag*U; environmental stress response, like *psp*C and *tsa*D; nutrient and metal transport, for example, ABC transporters and *feo*B; and gene regulation, like *sen*X3, *tet*R, and *php*P. The presence of these COGs enables *B. adolescentis* to adhere to the intestinal epithelium, compete for resources, withstand hostile conditions, and regulate its gene expression in response to environmental changes. At the same time, the accessory genome of *B. adolescentis* appears to be highly dynamic and shaped by selective pressures, including ecological and potentially geographical factors, reflecting the ongoing evolution of the species within the gut microbiota.

In addition to genomic insights, metagenomic analyses revealed the presence of *B. adolescentis* in more than half of the 10,620 samples, with its prevalence and relative abundance varying across different geographic regions. These variations may reflect the influence of environmental and lifestyle factors, such as dietary habits, shaping gut microbial composition worldwide. Correlation analysis identified positive associations with 226 species and negative ones with 114, indicating broad ecological connectivity. Consistent positive correlations were found with *B. longum* and *Collinsella aerofaciens*, as well as with beneficial taxa such as *Akkermansia muciniphila*, *Faecalibacterium prausnitzii*, and several butyrate producers, known for their roles in gut health, such as production of SCFAs, immunomodulation, and stimulation of mucin production. Conversely, negative correlations were observed among members of the Bacteroidota phylum and opportunistic taxa, including *Escherichia*, *Clostridium symbiosum*, and *Streptococcus mitis*, with some exhibiting geographic specificity. Overall, *B. adolescentis* emerged as one of the most interactive bifidobacterial species in the human gut, following only *B. longum*, highlighting its potential role in sustaining a balanced and resilient microbiota.

Comparative genomics also revealed a complex evolutionary history. The genomic potential of the species appears to be continuously modified by selective pressures. These pressures may trigger GoF processes in genes that were previously lost, as seen with some COGs in the cloud genome data set. Regarding GoF events, HGT emerges as a significant evolutionary driver, given the presence of hcHTGs in more than 80% of the *B. adolescentis* genomes, enabling the distinction between different types of HGT events. The nature of the predicted donor taxa further suggests that these gene acquisitions occurred within the gut environment, indicating that this habitat may serve as a recurrent setting for such transfers. Some derive from ancient acquisitions probably linked to speciation, as in the case of *ect*G. In contrast, others are more recent GoF events, such as the CRISPR-Cas genes, whose occurrence appears to be genome- or cluster-specific. These findings indicate that the *B. adolescentis* CRISPR-Cas loci likely originated from HGT and represent non-native elements that have been shaped by both evolutionary history and ecological context. The coexistence of Type I and Type II CRISPR-Cas systems within the species further underscores the dynamic nature of its mobilome, making this an intriguing aspect of *B. adolescentis* that merits deeper investigation in future studies.

Although the entire genetic potential of the species has been characterized, additional research is required to fully explore its implications. The data set can still be improved by adding genomes from underrepresented macro-regions. Furthermore, functional classification of many genes should be verified in future *in vitro* studies to validate their predicted domains and confirm their specificity at both inter- and intra-specific levels.

In conclusion, the highly conserved and unique metabolic capabilities of the species, combined with the plasticity of the accessory genome, resulting from a continuous process of adaptation to the environment, indicate that *B. adolescentis* is a species adapted for the colonization and proliferation in the human intestinal environment, as well as for the interaction with other microorganisms inhabiting the human gut microbiota.

## MATERIALS AND METHODS

### Genomic data set

A preliminary data set was created by collecting 2,244 genome assemblies of *B. adolescentis* from metagenomic sequencing and 873 genomes from isolated cultures of *B. adolescentis*. MAGs were obtained from various sources: 1,079 from Sanger Institute studies, 147 from the NCBI genome database, and 207 from the AWI-Gen 2 Microbiome Project. Furthermore, 811 MAGs were assembled within the framework of this project from publicly available human gut metagenome libraries. Moreover, 106 genome sequences from isolated cultures were retrieved from the Sanger Institute, 22 from the Probiogenomics laboratory collection, and 745 from the NCBI genome database.

Assemblies with a genome completeness level below 95% and a contamination level higher than 5% were identified and discarded using CheckM2 (15). An ANI value was then assigned to each genome to verify its classification as belonging to the species *B. adolescentis* and to discard redundant genomes showing an ANI value ≥99.99% when compared to any other genome in the data set (53, 54). The dereplication of genomes within the data set was performed by dRep (16), which utilized an ANI value of 99.99% to identify different taxonomic species subunits. The resulting data set includes 1,682 unique genomes belonging to the *B. adolescentis* species, as reported in Table S1 in the supplemental material.

We furthermore downloaded the genome sequences of type strains of the species present in the genus *Bifidobacterium*, all annotated complete bifidobacterial genomes available on NCBI of other species colonizing the human host, and, finally, all available genomes of the species in the genus present in the *B. adolescentis* phylogenetic group.

### Genome assembly

Genome assemblies were performed on local sequencing data of *B. adolescentis* isolates and using public shotgun metagenomic data of the human gut microbiota. Reads from strains isolated locally in the Probiogenomics laboratory were assembled using the MEGAnnotator2 software, while the METAnnotatorX2 tool was used to reconstruct *B. adolescentis* genomes from metagenomic samples of the human gut microbiota. SPAdes version 4.3.0 was used in both pipelines, invoking either “--isolate” or the “--meta” option, depending on the sample. For both software tools, the default parameters were utilized (55, 56).

### Pangenome analyses

Pangenome analyses were conducted using the software Roary (57), which enabled the dissection of the core genome (genes present in 99% to 100% of the assessed genomes), the soft-core genome (genes present in 95% to 99% of all genomes analyzed), and the accessory genome (genes present in less than 95% of all genomes in our data set) based on PROKKA annotation (58–60). The identity threshold for the constitution of Roary’s COGs varied depending on the data set, using 99% identity for monospecific data sets and 80% for data sets containing different species.

Using Roary’s output and a custom Python script, Heaps’ law was applied to calculate the state of the pangenome. One thousand different permutations of genome addition were computed, and the average progression of the pangenome was calculated. Genome annotation was performed using PROKKA. For proteins without specific functional annotation, such as hypothetical encoding proteins, a functional domain search was conducted using InterProScan with the Pfam 37.2 database (61, 62), with all other parameters set to their defaults. Additional databases were used to screen for specific bacterial features, such as IS Finder (identity cutoff of 70%) (63), BAGEL (identity cutoff 70%) (64), NCBI viruses genomes database (using blastn-short parameter) (65), and the bifidoprophage database (identity cutoff 50%) (66).

### Phylogenetic analyses

Phylogenetic distances were calculated using three different approaches to perform clustering within the data set. First, the multi-alignment of the core genome genes served as input for RAxML software (67) to calculate the phylogenetic distance between genomes based on single-nucleotide polymorphisms of the shared genes in the data set. RAxML was also employed to determine the phylogenetic distances of the genomes based on the presence/absence pattern of the accessory genome genes. Furthermore, phylogenetic distances were calculated based on the total average nucleotide identity (tANI) using the Gosselin 2022 protocol (68). Finally, phylogenetic distances based on the accessory genome were calculated using the accessory gene presence matrix obtained as the output of Roary.

### Cluster validation

Phylogenetic clustering was validated using two different approaches. The gANI matrix, also obtained with the Gosselin protocol, was used to obtain a distance matrix calculated as “1 − gANI.” This new distance matrix was utilized by the software OriginPro (OriginPro, version 2023b, Northampton, MA, USA) to calculate the optimal number of clusters using the elbow method. Then, HCA was conducted in OriginPro using the following parameters: cluster method, group average; distance type, Euclidean; standard variables, none; and cluster assembled by the sum of the squared distances. The use of “1 − gANI” distances, and not of tANI, and the non-normalization of distances in the HCA allowed us to correctly consider the slight variations in sequence identity (and therefore distances) present in the monospecific data set without amplifying them at the same time.

Genome clustering based on accessory gene content was performed using a custom Python script, employing Jaccard distances calculated from a binary gene presence/absence matrix. The distances obtained were used to determine the optimal number of clusters using the elbow method and to actualize the subdivision through HCA. A PCoA was used to visualize the distribution of the clusters in a Cartesian space. The impact of all accessory genes on the PCoA was calculated to identify genes with a statistically significant influence. The Z-score and *P*-value, corrected by the Benjamini-Hochberg method (*P*-value BH), were calculated from these. Genes with a *P*-value BH < 0.05 were considered significant.

To obtain the sequences of the genes influencing the PCoA, the genomes deriving from biological strains/isolates, with the best quality (contamination and completeness calculated with CheckM2), whose minimum combination contains all of them, were identified. Sequences identified as “hypothetical proteins” were compared to the InterPROscan database.

### Metagenomic correlation analysis

To identify correlations between bifidobacterial species and other microbial taxa, taxonomic profiles of 10,620 shotgun metagenomic samples belonging to 65 BioProjects were analyzed. These samples had been pre-selected to include only data sets generated using Illumina sequencing platforms and derived exclusively from healthy adult subjects, thereby minimizing potential confounding effects related to sequencing technology, host age, or health status.

Microbial profiles were generated using the METAnnotatorX2 software, ensuring robust taxonomic classification (56). Prior to profiling, reads were quality-filtered and filtered to remove adapters, human-derived sequences, and ribosomal gene sequences. Metadata on the age and geographic origin of each sample were retrieved from NCBI (Table S5).

To investigate the relationship between *B. adolescentis* and other microbial taxa, Spearman correlation analysis was conducted using a custom Python script, which utilized the relative abundance and prevalence of bacterial taxa, adjusting *P*-values for multiple testing using the Benjamini-Hochberg (FDR) corrections (Table S6). Furthermore, geographic data were used to assess whether the significance of the observed correlations was consistent across the entire data set or driven by one or more macro-regions.

### HGT events and modification of the functional potential of the *B. adolescentis* species

The possible non-native status was assigned to genes identified in the pangenome data set that presented a CUB and a GC content significantly different from other genes (69). Calculation of the CUB factors and GC content was performed using a custom Python script. Three different factors were considered in the CUB: relative synonymous codon usage (RSCU), which is the codon usage compared to the theoretical maximum; effective number of codons (ENC), indicating the specificity required by the gene in the codon usage; and the codon adaptation index (CAI), which considers the codon usage compared to highly expressed reference genes. The thresholds of these four factors were calculated dynamically, considering the deviation from the factors’ values in the other coding genes present in the genome. Each factor is considered TRUE or FALSE if the behavior of the gene for that factor deviates from those of at least 90% of the genes present in the genome (value above the 90th percentile for GC, RSCU, and ENC content and below the 10th percentile for CAI). Genes with all four TRUE factors were therefore deemed as high-confidence horizontally transferred genes (hcHTGs). To identify putative hcHTG donors, a blastp search was performed against the NCBI amino acid database, excluding matches to proteins derived from *Bifidobacterium* species.

To study the gain and loss of function, the gene presence matrix of Roary and the phylogenetic tree of the core genome of a second data set, composed of genomes of *B. adolescentis* and type strains of the *B. adolescentis* group, were used to evaluate the global process of acquisition and loss of functions. To achieve this, the genomes of the type strains of the clade were added to the *B. adolescentis* genome data set. This new data set was re-annotated by PROKKA, and the pangenome analyzed by Roary, with an 80% identity threshold set for the formation of COGs. For each species, the pangenome formed by all available genomes in the NCBI database was calculated by Roary, with a 90% identity threshold. Events of gain or loss of function were calculated using a custom Python script, which applied Wagner parsimony with a gain penalty of 2. The statistical significance of GoF and LoF events was calculated using a cross-validation system (70).

## Data Availability

All the new genome sequences from isolated cultures were deposited and are available in NCBI under the projects PRJNA1011865 and PRJNA1301581. Genomes assembled from metagenomic samples were deposited in Zenodo under the identification number 16743655. All publicly available metagenomic data sets supporting the findings of this study can be obtained through the accession code reported in the supplemental material.

## SUPPLEMENTAL MATERIAL

Supplemental figures msystems.01173-25-s0001.pdf Fig. S1 and S2.

Supplemental tables msystems.01173-25-s0002.xlsx Tables S1-S11.

ASM does not own the copyrights to Supplemental Material that may be linked to, or accessed through, an article. The authors have granted ASM a non-exclusive, world-wide license to publish the Supplemental Material files. Please contact the corresponding author directly for reuse.
