# Supplementary material for: Assessment of genome evolution in Bifidobacterium adolescentis indicates genetic adaptation to the human gut
Source: mSystems. 2026 Feb 6;11(3):e01173-25. doi: 10.1128/msystems.01173-25 (PMC13011404; doi:10.1128/msystems.01173-25)
Supplement: Supplemental figures — Fig. S1 and S2. [file msystems.01173-25-s0001.pdf]

Supplementary Figure 1

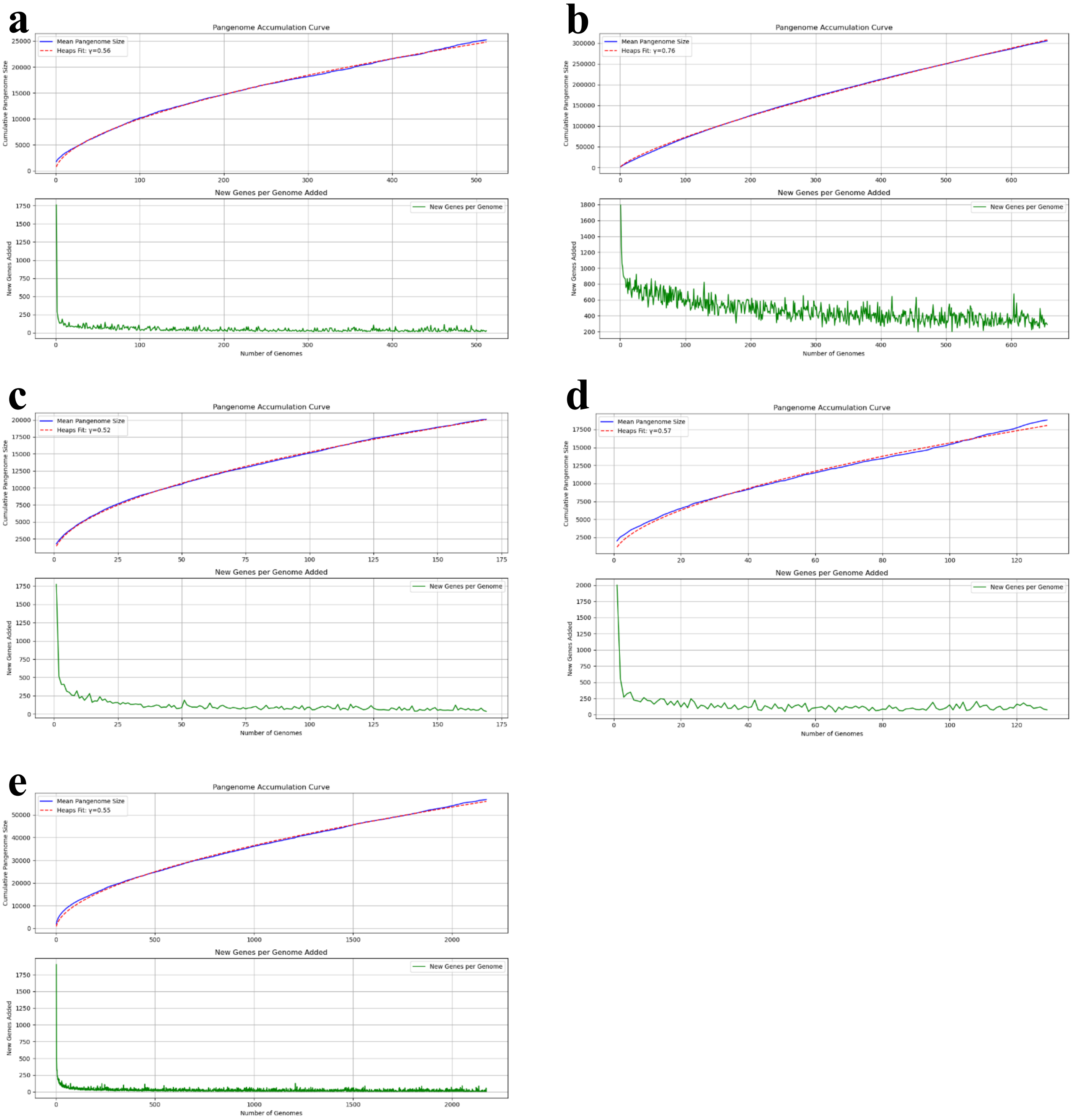

Supplementary Figure 1. Pangenome growth of human-associated *Bifidobacterium* species. Pangenome accumulation curves were generated from high-quality genomic sequences retrieved from NCBI for five *Bifidobacterium* species commonly associated with the human host: *(a) B. bifidum*, *(b) B. breve*, *(c) B. catenulatum*, *(d) B. dentium*, and *(e) B. longum*. For each species, two plots are shown. The top panels illustrate the overall pangenome growth, depicting the cumulative number of genes (y-axis) as a function of the number of genomes sequentially added (x-axis). The bottom panels display the average number of newly identified genes with each additional genome, highlighting the rate of gene discovery and the openness of the species' pangenome. All analyses were performed using high-quality genome assemblies, ensuring consistency across datasets and comparability with the *B. adolescentis* pangenome.

Supplementary Figure 2

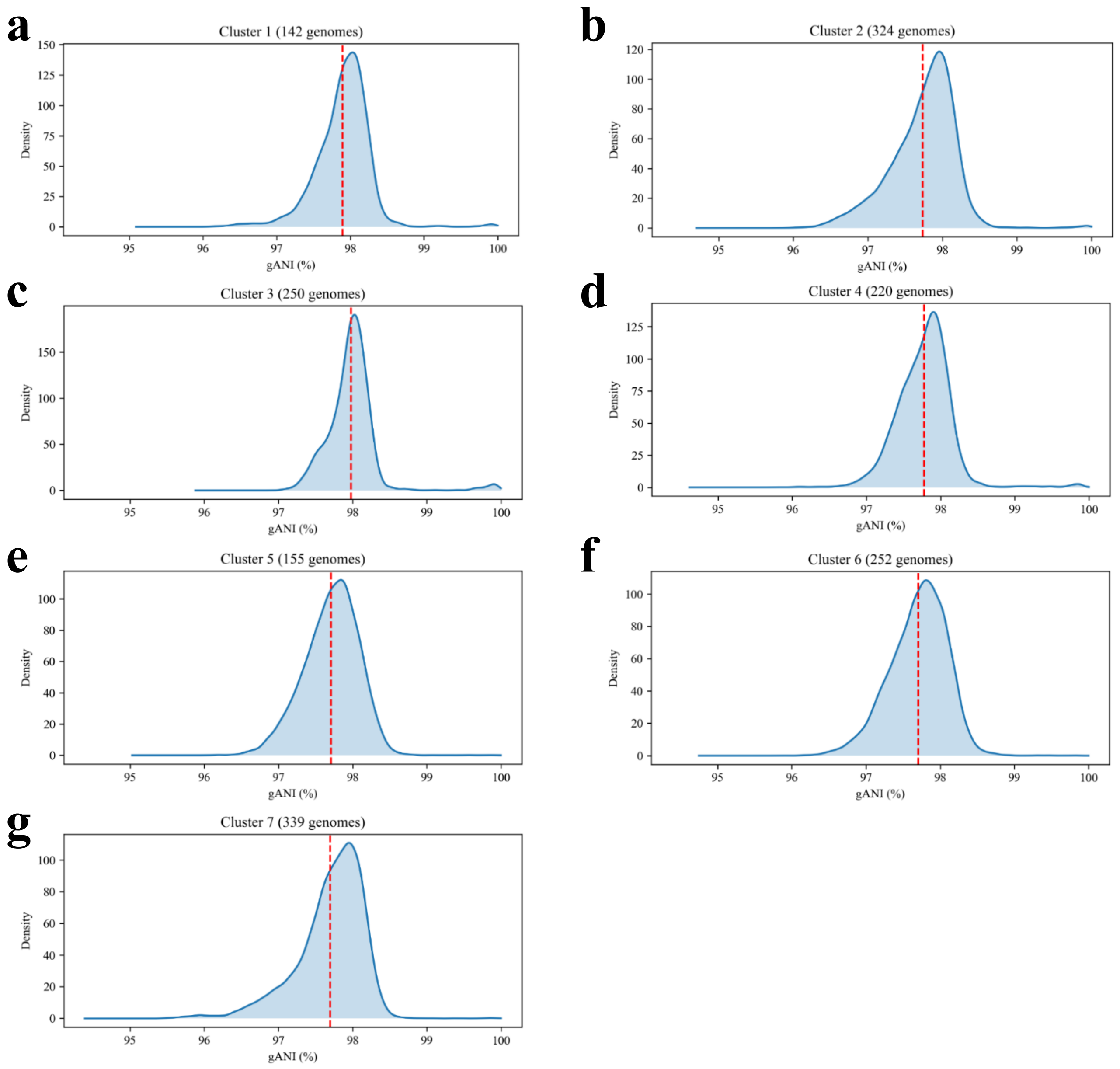

Supplementary Figure 2. Genetic distances within *B. adolescentis* genomic clusters. Each panel (from a to g) represents a density plot of ANI values obtained from pairwise genome comparisons across the seven *B. adolescentis* genomic clusters. The mean ANI value and range are indicated for each cluster.
